# Differential expression of Na^+^/K^+^/Cl^−^ cotransporter 1 in neurons and glial cells within the superficial spinal dorsal horn of rodents

**DOI:** 10.1038/s41598-020-68638-3

**Published:** 2020-07-16

**Authors:** Fariba Javdani, Krisztina Hegedűs, Camila Oliveira Miranda, Zoltán Hegyi, Krisztina Holló, Miklós Antal

**Affiliations:** Department of Anatomy, Histology and Embryology, Faculty of Medicine, University of Debrecen, Nagyerdei krt. 98, Debrecen, 4032 Hungary

**Keywords:** Cell biology, Pain

## Abstract

Although convincing experimental evidence indicates that Na^+^/K^+^/Cl^−^ cotransporter 1 (NKCC1) is involved in spinal nociceptive information processing and in the generation of hyperalgesia and allodynia in chronic pain states, the cellular distribution of NKCC1 in the superficial spinal dorsal horn is still poorly understood. Because this important piece of knowledge is missing, the effect of NKCC1 on pain processing is still open to conflicting interpretations. In this study, to provide the missing experimental data, we investigated the cellular distribution of NKCC1 in the superficial spinal dorsal horn by immunohistochemical methods. We demonstrated for the first time that almost all spinal axon terminals of peptidergic nociceptive primary afferents express NKCC1. In contrast, virtually all spinal axon terminals of nonpeptidergic nociceptive primary afferents were negative for NKCC1. Data on the colocalization of NKCC1 with axonal and glial markers indicated that it is almost exclusively expressed by axon terminals and glial cells in laminae I–IIo. In lamina IIi, however, we observed a strong immunostaining for NKCC1 also in the dendrites and cell bodies of PV-containing inhibitory neurons and a weak staining in PKCγ-containing excitatory neurons. Our results facilitate further thinking about the role of NKCC1 in spinal pain processing.

## Introduction

Cation-chloride-cotransporters are crucially important in the regulation of intracellular and extracellular chloride concentrations. Although there are nine members of the cation-chloride cotransporter family^1–3^, Cl^−^ gradient across the cell membranes of neurons is controlled by only two such proteins: K^+^/Cl^−^ cotransporter 2 (KCC2) and Na^+^/K^+^/2Cl^−^ cotransporter 1 (NKCC1)^4–6^. KCC2 extrudes chloride from the cytosol, whereas NKCC1 moves chloride ions into the cells^7–11^. Thus, NKCC1 is responsible for the intracellular accumulation of chloride^12,13^ and, acting alone or in an antagonistic relationship with KCC2, it sets the equilibrium potentials for GABA_A_ and glycine receptor channels^3^. By serving as a primary regulator of the hyperpolarizing or depolarizing effects of GABA_A_ and glycine receptor activation, NKCC1 acts as one of the key players in shaping complex neural network activity^14,15^, including spinal nociceptive information processing^6,16–19^.

Although the pro-nociceptive role of NKCC1 in spinal pain processing has been convincingly demonstrated^4,6,20^, its effect on hyperalgesia and allodynia at the cellular level is still open to conflicting interpretation. The reason for this ambiguity is that inconsistent and contradictory results prevent generalization from being made about the cellular distribution of NKCC1. There is no general agreement on whether NKCC1 in the spinal cord is expressed by neurons and/or glial cells^21,22^. There are also contradictory results regarding whether this protein is distributed in all primary afferent neurons^23–25^ or only in certain subsets of them^26^. Despite much convincing evidence that at least some neurons in dorsal root ganglia express NKCC1, there have been no reports on the localization of the transporter in the spinal axon terminals of nociceptive afferents. We intended to contribute to this debate and provide experimental data on which the pro-nociceptive role of NKCC1 as well as its effect on hyperalgesia and allodynia can be more accurately interpreted.

Thus, by using a very specific and highly sensitive antibody against NKCC1, we investigated the neuronal and glial localization of NKCC1 in the nociceptive recipient layers (laminae I–II) of the spinal dorsal horn by immunohistochemical techniques. Our results provide a set of valuable new data about the neuronal and glial localization of NKCC1 within the superficial spinal dorsal horn and thus facilitate further thinking about the role of NKCC1 in spinal pain processing.

## Results

### Specificity of the NKCC1 antibody

**Figure 1:**
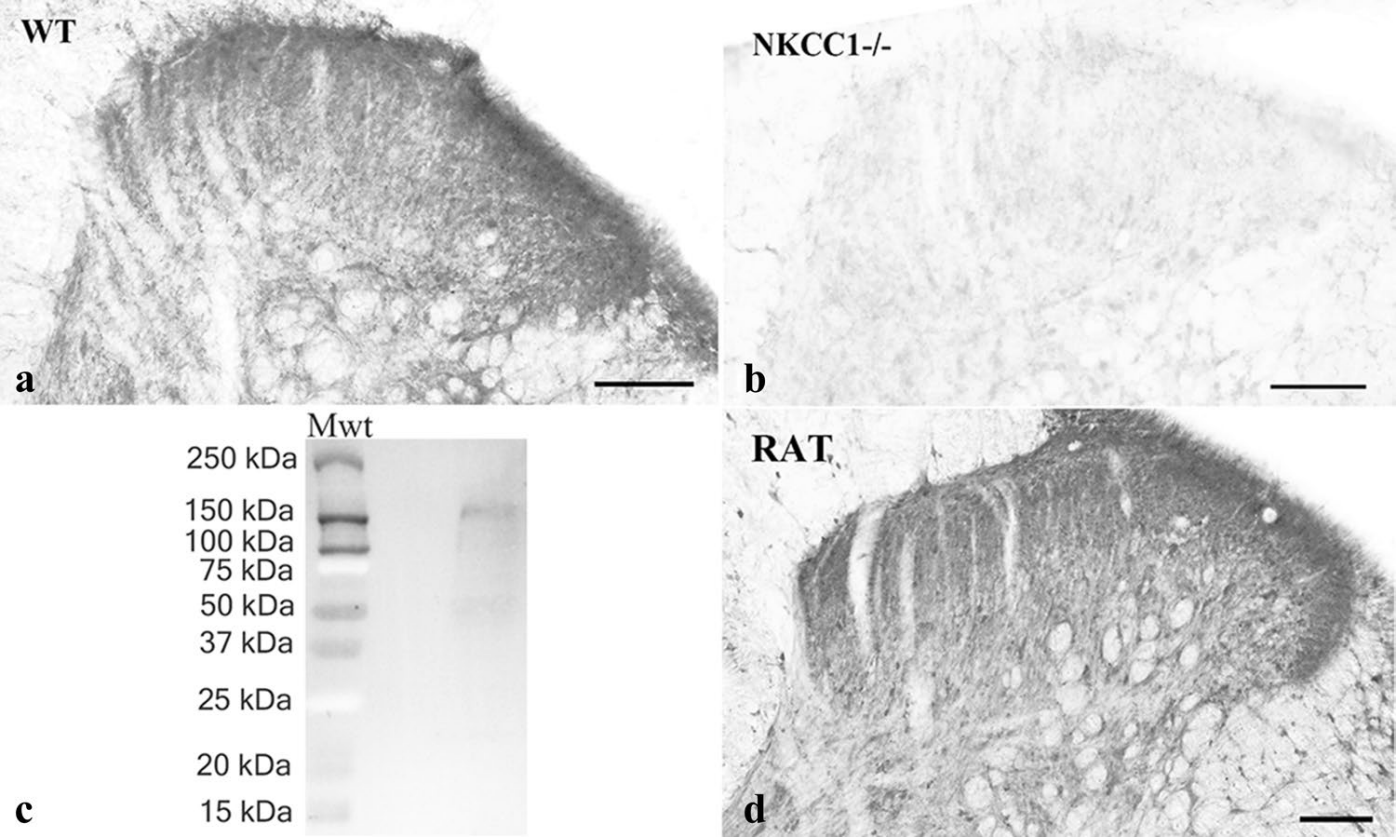
Specificity of the anti-NKCC1 antibody and distribution of NKCC1 immunoreactivity in the spinal dorsal horn. **a–b**. Photomicrographs showing immunoreactivity for NKCC1 in wild-type (**a**) and knockout (**b**) mice. NKCC1 immunostaining can be observed in the dorsal horn of the wild-type mouse, while the immunoreactivity is completely abolished from the dorsal horn of the NKCC1 knockout animal. **c**. Western blot analysis reinforces the specificity of the anti-NKCC1 antibody. The single immunoreactive band on the full-length running gel indicates that the antibody detects a protein with a molecular mass of ~ 160 kDa that corresponds to the molecular mass of NKCC1. For the molecular weight calibration, the precision plus protein dual color standards were used on which the blue and red colors appear as gray and white, respectively, after the black and white conversion (Bio-Rad, Hercules, California, USA) **d**. Photomicrographs showing immunoreactivity for NKCC1 in the dorsal horn of the rat spinal cord. Scale bars: 100 µm.

To test the specificity of the antibody raised against NKCC1, we stained sections obtained from the spinal cord of NKCC1 knockout and wild type mice, and carried out a Western blot analysis. No specific staining was observed in sections obtained from NKCC1 knockout mice (Fig. 1b). The dorsal horn of wild type mice, however, was heavily stained, and the pattern of immunostaining was similar to that observed in the rat (Fig. 1 a, d). The Western blot analysis showed only one immunostained band at the molecular weight of the glycosylated NKCC1 protein (~ 160 kDa; Fig. 1c).

**Figure 2:**
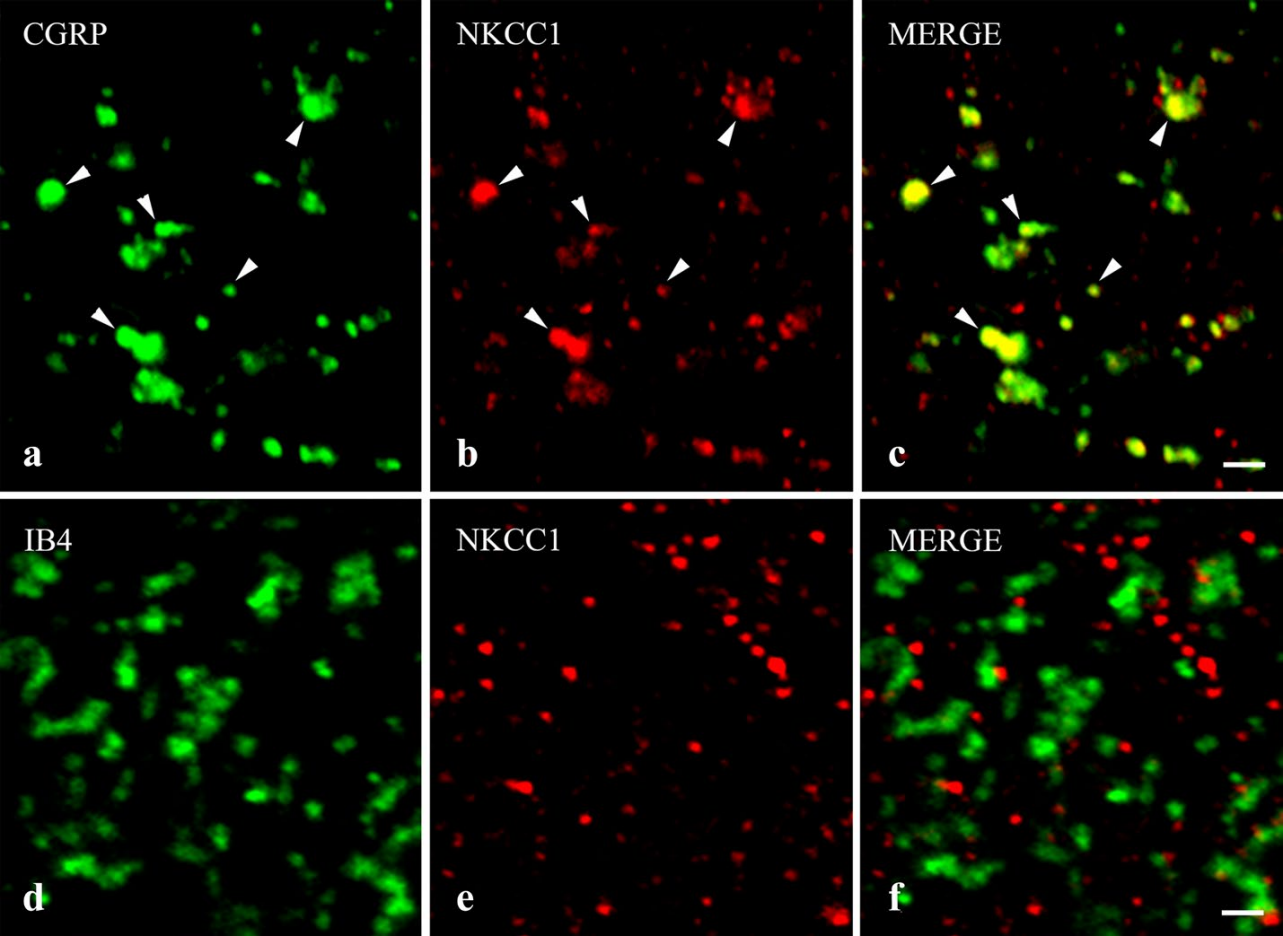
Immunostaining for NKCC1 in spinal axon terminals of nociceptive primary afferents. Micrographs of 1 µm thick laser scanning confocal optical sections illustrating the colocalization between immunolabeling for NKCC1 (red; **b**, **e**) and immunoreactivity for markers that are specific for axon terminals of peptidergic (CGRP, green; **a**) and nonpeptidergic (IB4-binding, green; **d**) nociceptive primary afferents in the superficial spinal dorsal horn. Mixed colors (yellow) on the superimposed images (**c**, **f**) indicate double labeled structures. Some double-stained varicosities are marked with arrowheads. Note that NKCC1 immunoreactivity can be observed in most of the peptidergic and almost none in the nonpeptidergic axon terminals of nociceptive primary afferents. Scale bar: 2 µm.

**Figure 3:**
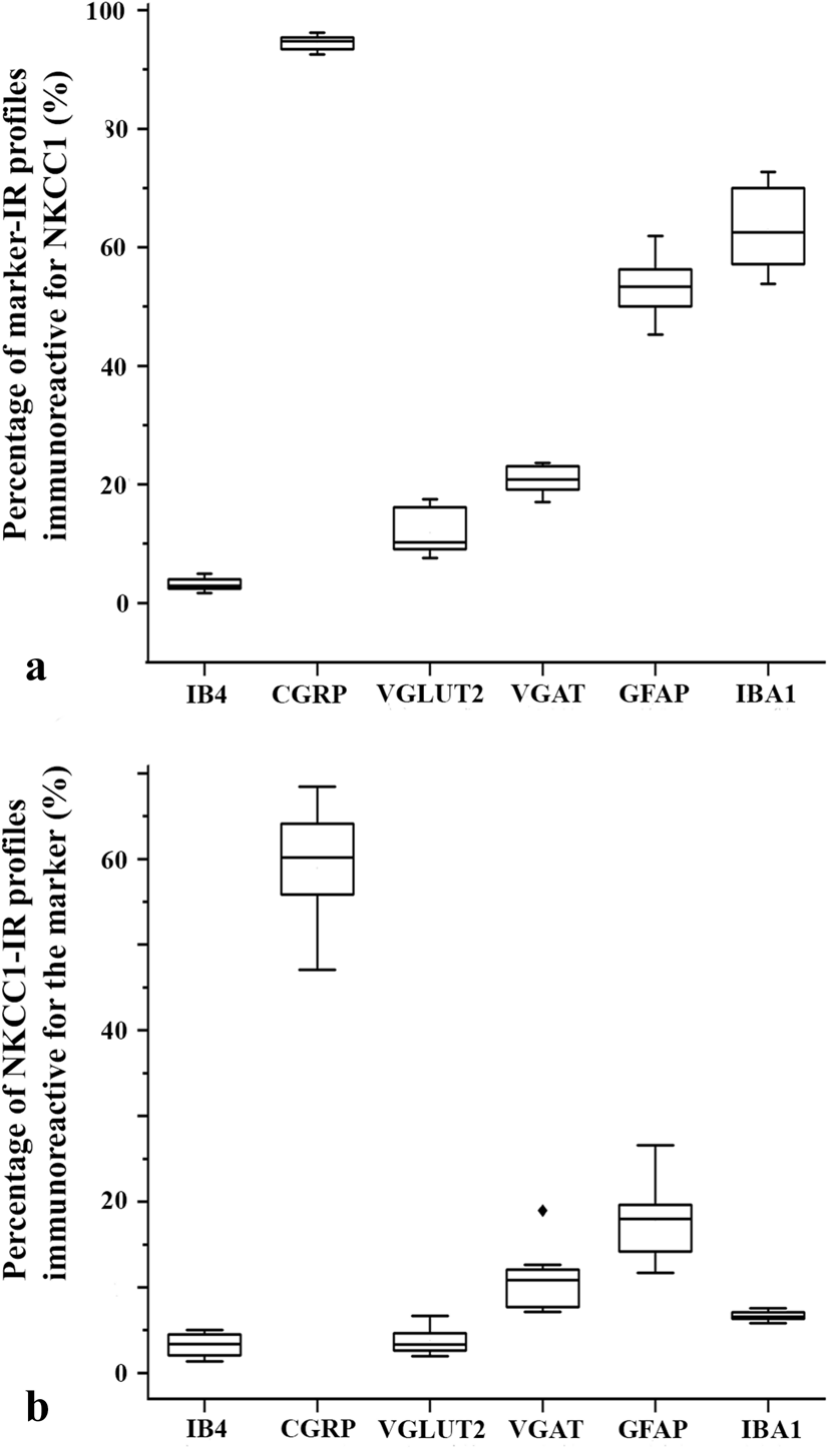
Box plot histograms showing the degree of colocalization between immunoreactivity for NKCC1 and selected axonal and glial markers in laminae I–II of the spinal dorsal horn. (**a**) Percentage of profiles immunoreactive for the applied axonal and glial markers that were also labeled for NKCC1. (**b**) Percentage of profiles immunoreactive for NKCC1 that were found within the confines of areas immunostained for the applied axonal and glial markers.

**Figure 4:**
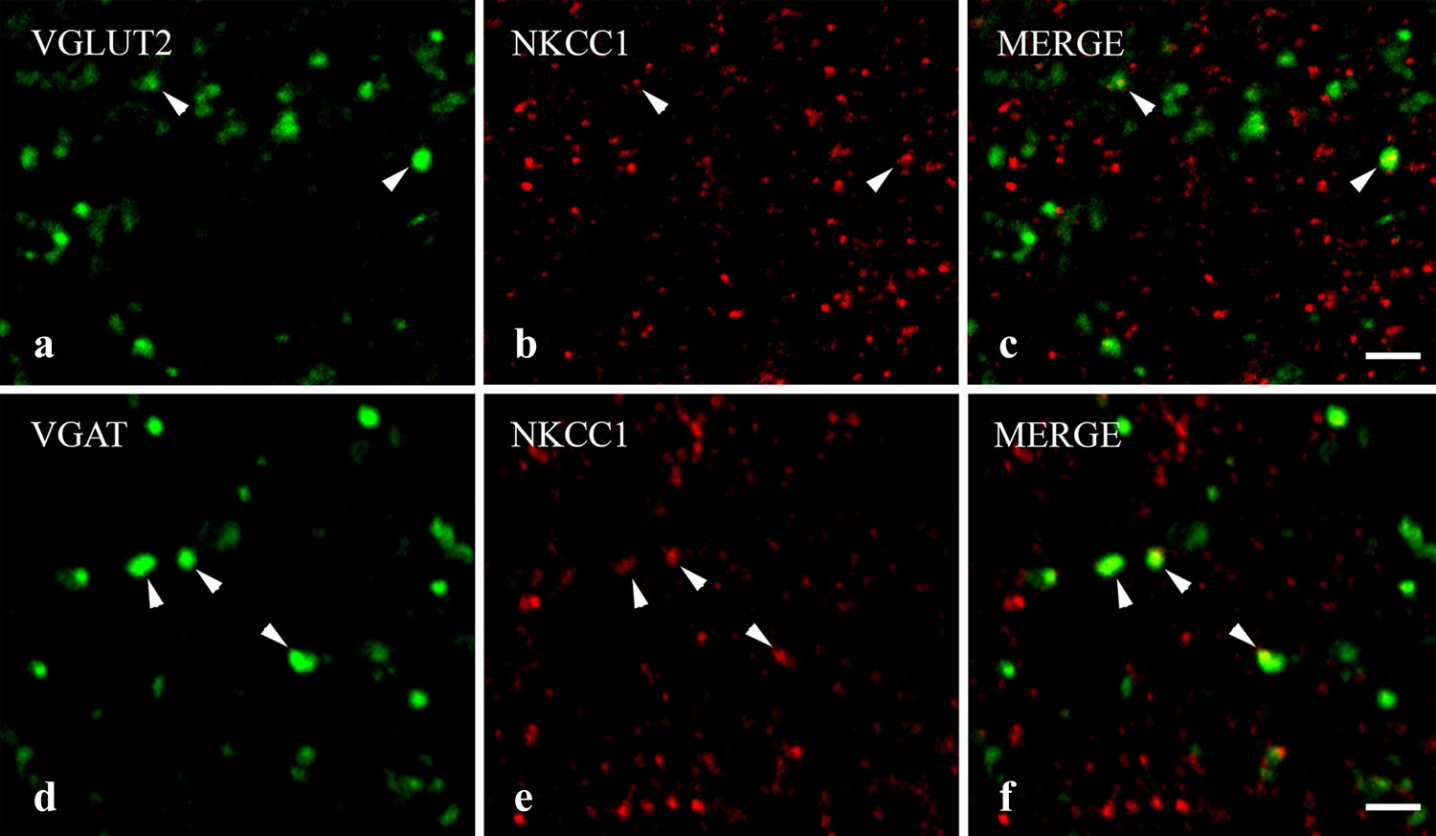
Immunostaining for NKCC1 in axon terminals of spinal excitatory and inhibitory neurons. Micrographs of 1 µm thick laser scanning confocal optical sections illustrating the colocalization between immunolabeling for NKCC1 (red; **b**, **e**) and immunoreactivity for markers that are specific for excitatory (VGLUT2, green; (**a**) and inhibitory (VGAT, green; **d**) axon terminals of intrinsic neurons in the superficial spinal dorsal horn. Mixed colors (yellow) on the superimposed images (**c**, **f**) indicate double-labeled structures. Double-stained varicosities are marked with arrows. Scale bar: 2 µm.

### Distribution of NKCC1 immunostaining in the dorsal horn of the spinal cord

We observed a strong immunostaining for NKCC1 in the lumbar spinal cord of rats. Punctate immunostained profiles were distributed throughout the dorsal horn but the staining was more intense in laminae I–II than that in deeper laminae (Fig. 1d). Glial-like immunostained profiles were also observed in the white matter (Fig. 1d).

### Expression of NKCC1 in neurons and glial cells

#### Colocalization of NKCC1 with markers of nociceptive primary afferents

On the basis of their neurochemical characteristics, nociceptive primary afferents are divided into peptidergic and nonpeptidergic subgroups in the superficial spinal dorsal horn^27^. Most of the peptidergic ones contain calcitonin gene-related peptide (CGRP), whereas the nonpeptidergic afferents selectively binds isolectin-B4 (IB4)^27^. Thus, to reveal the expression of NKCC1 on nociceptive primary afferent terminals in the spinal cord, we studied the colocalization of NKCC1 with CGRP and IB4-binding.

Confirming previous results, laminae I–IIo showed a strong immunostaining for CGRP^28,29^, and most of the CGRP immunostained boutons were also immunoreactive for NKCC1. We found that 60.25 ± 1.88% of NKCC1-IR puncta were located in peptidergic axon terminals stained for CGRP, whereas 94.45 ± 0.43% of CGRP-IR terminals were also immunostained for NKCC1 (Figs. 2a–c, 3).

As previously reported, IB4-binding resulted in a dense punctate labeling in lamina IIi^30^. Concerning the colocalization between NKCC1 immunostaining and IB4-binding, we found that only 3.09 ± 0.47% of NKCC1-IR puncta were in nonpeptidergic axon terminals binding IB4, and 3.10 ± 0.36% of IB4-binding nonpeptidergic axon terminals were positive for NKCC1 (Figs. 2d–f, 3).

#### Colocalization of NKCC1 with markers of axon terminals of glutamatergic and GABAergic spinal neurons

Vesicular glutamate transporter 2 (VGLUT2) in immunofluorescence studies can be used as a marker for axon terminals of intrinsic excitatory spinal neurons^31–33^, whereas GABAergic and glycinergic inhibitory interneurons transport both GABA and glycine into synaptic vesicles with the aid of the vesicular GABA transporter (VGAT)^34,35^. Therefore, to study the expression of NKCC1 on axon terminals of excitatory, glutamatergic and inhibitory, GABAergic and/or glycinergic spinal neurons we investigated the colocalization between NKCC1 and VGLUT2 as well as VGAT immunoreactivity.

As expected, VGLUT2-IR profiles were evenly scattered in laminae I–II^31–33^. Despite the strong staining obtained for both NKCC1 and VGLUT2, the colocalization between the two molecules was low. Only 3.77 ± 0.52% of NKCC1-IR puncta were found in axon terminals immunostained for VGLUT2, and 11.87 ± 1.31% of VGLUT2 profiles were also immunostained for NKCC1 (Figs. 3, 4c).

Similar to earlier reports^34^, VGAT-IR puncta were densely and evenly scattered throughout the superficial spinal dorsal horn. We recovered more NKCC1-IR puncta in VGAT-positive axon terminals than in those immunostained for VGLUT2, but the colocalization between NKCC1 and VGAT was still moderate. We found that 10.51 ± 1.26% of NKCC1-IR puncta in axon terminals were stained for VGAT, whereas NKCC1-IR puncta were recovered in 20.76 ± 0.82% of VGAT-IR axon terminals (Figs. 3, 4c,d).

#### Colocalization of NKCC1 with glial markers

Increasing experimental evidence indicates the role of astrocytes and microglial cells in spinal pain processing^36–39^. Thus, we studied the colocalization of NKCC1 with astrocytic and microglial markers; with glial fibrillary acidic protein (GFAP) and ionized calcium-binding adaptor molecule 1 (IBA1), respectively^40^.

**Figure 5:**
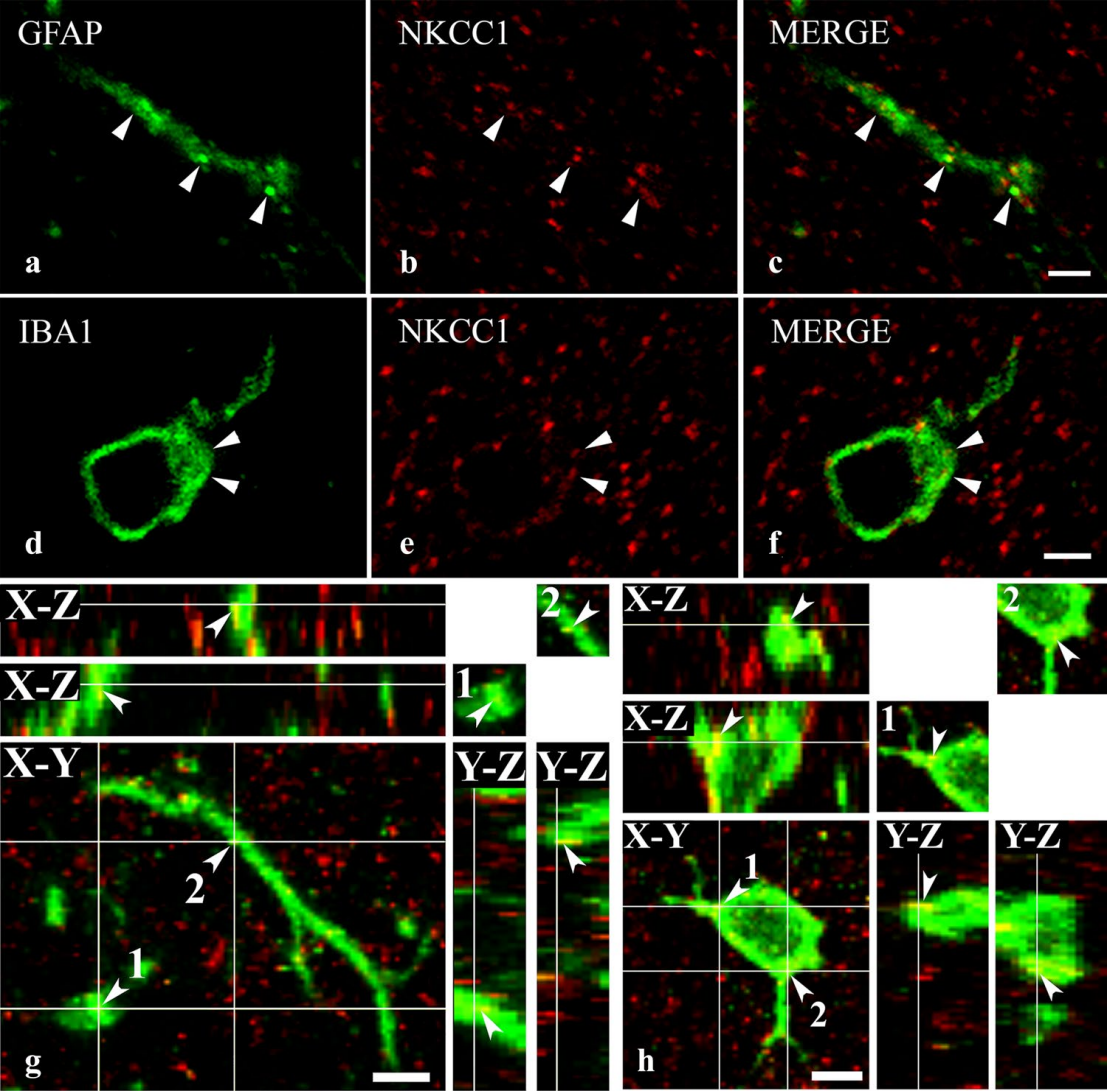
Immunostaining for NKCC1 in astrocytes and microglial cells. Micrographs of 1 µm thick laser scanning confocal optical sections illustrating the colocalization between immunolabeling for NKCC1 (red; **b**, **e**) and immunoreactivity for markers that are specific to astrocytes (GFAP, green; **a**) and microglial cells (IBA1, green; **d**) in the superficial spinal dorsal horn. Mixed colors (yellow) on the superimposed images (**c**, **f**) indicate double-labeled structures. Micrographs of short series of confocal optical sections double immunostained for NKCC1 (red) and GFAP (green) or IBA1 (green) show the colocalization between NKCC1 and GFAP (**g**) as well as IBA1 (**h**) illustrated in X–Y, X–Z and Y–Z projections. Two selected points (labeled with 1 and 2) of colocalization between the markers are at the crossing point of two lines indicating the planes through which orthogonal views of X–Z and Y–Z projections were drawn. Small inserts in the upper right corner of both g and h show the selected two points in X–Y dimension without the lines. The corresponding X–Z and Y–Z projections are beside and below these inserts. According to the orthogonal images, as it is indicated by the mixed color (yellow), NKCC1 immunostained puncta 1 and 2 are within the confines of the GFAP as well as the IBA1 immunoreactive profiles. Double stained spots are marked with arrows. Scale bar: 5 µm (**a**–**f**), 2 µm (**g**, **h**).

More than half of the investigated glial cells expressed NKCC1, but the density of spots immunostained for NKCC1 was sparse on both types of glial cells. We found that 53.23 ± 1.84% of GFAP-IR profiles were immunoreactive for NKCC1, but only 16.86 ± 1.67% of NKCC1-IR puncta were scattered over profiles stained for GFAP (Figs. 3, 5a–c,g). Similar to GFAP, 62.99 ± 2.46% of IBA1-IR profiles were immunostained for NKCC1. However, only 6.85 ± 0.25% of NKCC1-IR puncta were recovered in profiles stained for IBA1 (Figs. 3, 5d–f,h).

#### Colocalization of NKCC1 with parvalbumin and protein kinase C gamma

The colocalization data of NKCC1-IR puncta in lamina I–IIo, where the peptidergic (CGRP-IR) nociceptive primary afferents terminate^28,29^, showed that NKCC1 is almost exclusively expressed by axon terminals and glial cells, primarily due to the remarkably high expression of NKCC1 in CGRP-IR axon terminals. In lamina IIi, where the nonpeptidergic (IB4-binding) nociceptive primary afferents terminate^30^, however, only a minor proportion of the IB4-binding axon terminals showed positive staining for NKCC1. Taking low axonal and moderate glial expression into consideration, we hypothesized that, in contrast to lamina I–IIo, the somatodendritic compartment of neurons may show strong expression for NKCC1 in lamina IIi. To test this notion, we studied the distribution of NKCC1-IR puncta on the dendrites of parvalbumin (PV)-containing inhibitory and protein kinase C gamma (PKCγ)-containing excitatory neurons, which are located in lamina IIi, and play major roles in spinal pain processing^41–43^.

**Figure 6:**
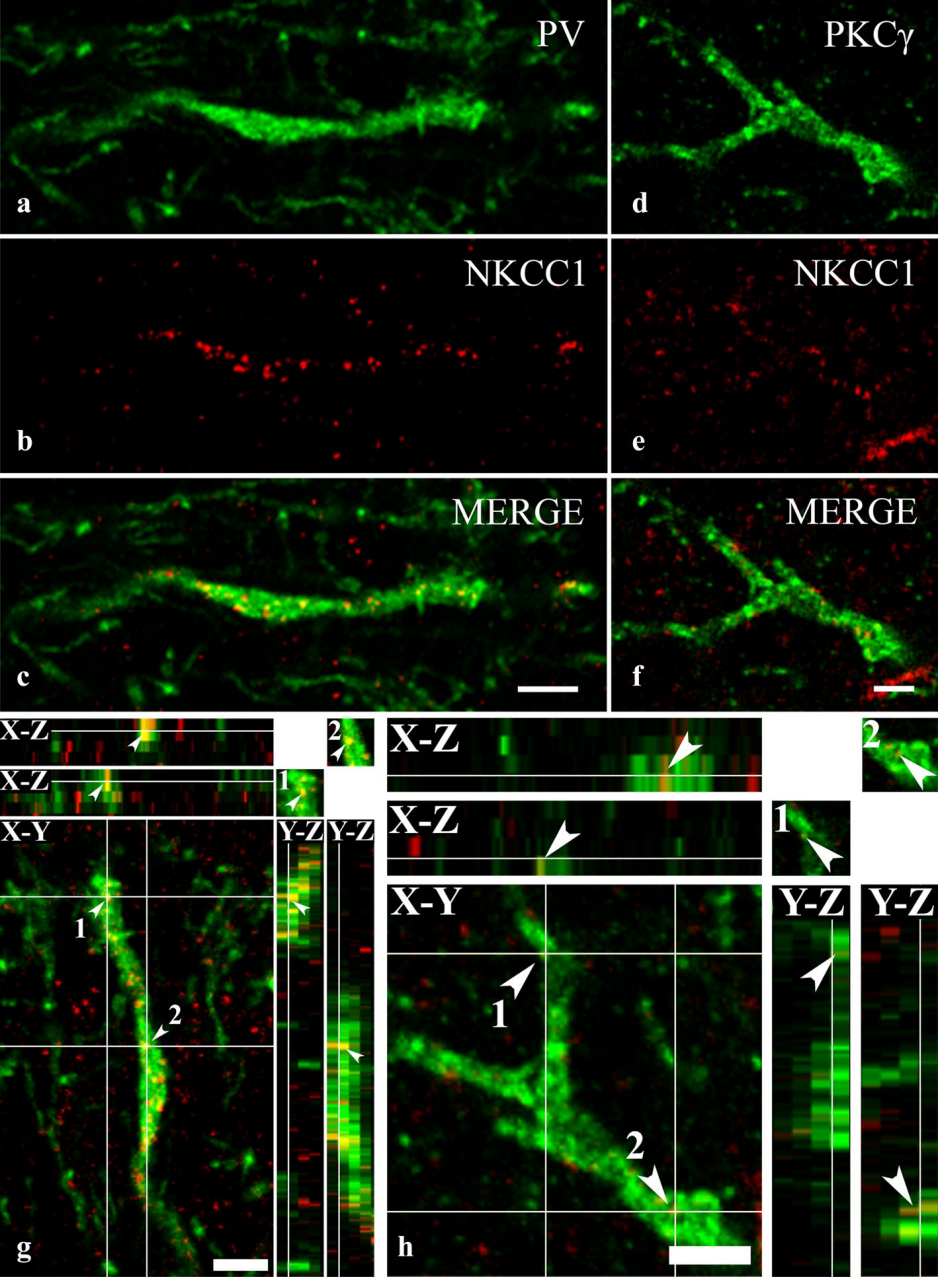
Immunostaining for NKCC1 in dendrites of spinal neurons containing PV and PKCγ. Micrographs of 1 µm thick laser scanning confocal optical sections double stained for PV (**a**) and NKCC1 (**b**) and for PKCγ (**d**) and NKCC1 (**e**). Mixed colors on the merged images (**c**, **f**) indicate that NKCC1 is strongly expressed by dendrites of PV-IR dendrites (**c**), whereas NKCC1-IR puncta are only sparsely scattered over the PKCγ-IR dendritic segment (**f**). Micrographs of short series of confocal optical sections double immunostained for NKCC1 (red) and PV (green) or PKCγ (green) show the colocalization between NKCC1 and PV (**g**) as well as PKCγ (**h**) illustrated in X–Y, X–Z and Y–Z projections. Two selected points (labeled with 1 and 2) of colocalization between the markers are at the crossing point of two lines indicating the planes through which orthogonal views of X–Z and Y–Z projections were drawn. Small inserts in the upper right corner of both g and h show the selected two points in X–Y dimension without the lines. The corresponding X–Z and Y–Z projections are beside and below these inserts. According to the orthogonal images, as it is indicated by the mixed color (yellow), NKCC1 immunostained puncta 1 and 2 are within the confines of the PV as well as the PKCγ immunoreactive profiles. Scale bars: 5 µm (**a**–**f**), 2 µm (**g**, **h**).

**Figure 7:**
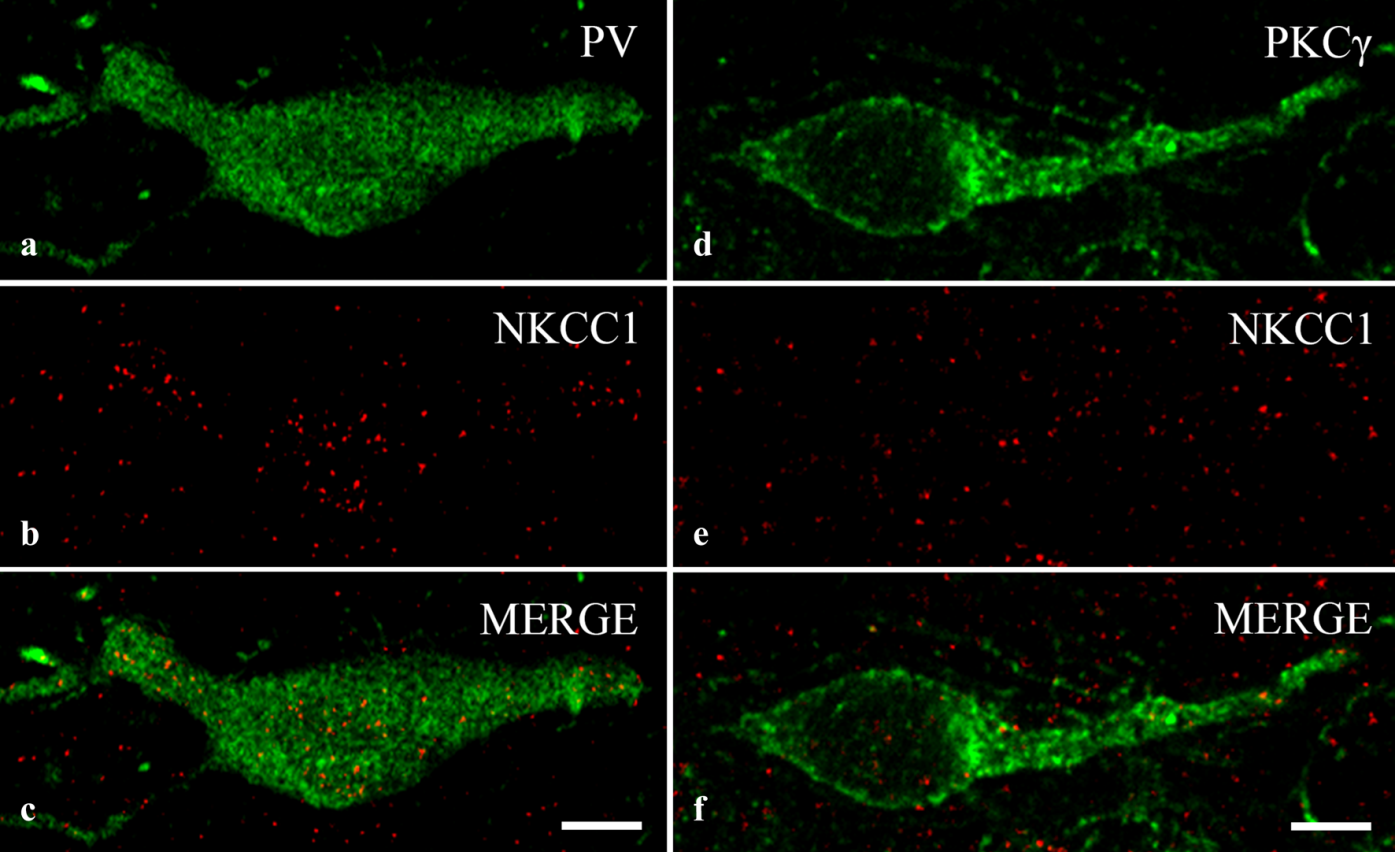
Immunostaining for NKCC1 in the cell bodies of spinal neurons containing PV and PKCγ. Micrographs of 1 µm thick laser scanning confocal optical sections double stained for PV (**a**) and NKCC1 (**b**) and for PKCγ (**d**) and NKCC1 (**e**). The merged images (**c**, **f**) show strong expression of NKCC1 in the cell body of the PV-IR neuron (**c**) and moderate staining for NKCC1 in the PKCγ-IR neuron (**f**). Scale bars: 10 µm.

Fulfilling our expectations, we recovered NKCC1-IR puncta in high numbers in the dendrites of PV-IR (Fig. 6a–c,g) neurons. However, immunostaining for NKCC1 was weak in PKCγ-IR dendrites (Fig. 6d–f,h). In addition to the dendrites, we observed robust immunostaining for NKCC1 in the cell bodies of PV-IR neurons (Fig. 7a–c). As in the dendrites, most of the immunostained puncta were scattered within the cytoplasm, and only a few of them were located so close to the border of the PV-immunostained areas that they could be regarded as cell membrane-associated staining (Figs. 6a–c,f, 7a–c). In contrast to the heavy staining of PV-IR neurons, PKCγ-IR neurons showed sparse immunostaining for NKCC1 not only in dendrites but also in the cell bodies (Fig. 7d–f).

In addition to PV-IR and PKCγ-IR neurons, other types of neurons may also contribute to the formation of the neuronal assembly of lamina IIi, and the dendrites as well as cell bodies of these neurons may also express NKCC1; therefore, we did not perform any quantitative analysis concerning the distribution of NKCC1-IR puncta in the PV-IR and PKCγ-IR neurons. However, on the basis of the colocalization data of NKCC1-IR puncta with axonal and glial markers we assume that approximately 60% of NKCC1 immunoreactivity might be confined to the dendrites and cell bodies of neurons in lamina IIi.

## Discussion

By studying the cellular expression of NKCC1 with immunohistochemical methods, we provided the first detailed account of the distribution of NKCC1 in neurons and glial cells in the superficial spinal dorsal horn of rats. We provide the first demonstration in the literature that almost all spinal axon terminals of CGRP-IR peptidergic nociceptive primary afferents in laminae I–IIo express NKCC1. In contrast, virtually all spinal axon terminals of IB4-binding nonpeptidergic nociceptive primary afferents in lamina IIi were negative for NKCC1. The colocalization data of NKCC1 indicated that it is almost exclusively expressed by axon terminals and glial cells in laminae I–IIo. In lamina IIi, however, we also revealed strong immunostaining for NKCC1 in the dendrites and cell bodies of selected neurons, which was stronger in PV-IR inhibitory cells and weaker in PKCγ-IR excitatory cells.

### Differential distribution of NKCC1 in neurons and glial cells

The expression of NKCC1 protein and mRNA in dorsal root ganglion (DRG) neurons has been well documented^12,19,23,25,44^. The experimental findings, however, are contradictory concerning the identification of subgroups of DRG neurons that are positive or negative for NKCC1. It has been reported that NKCC1 protein can be detected in virtually all DRG neurons in the frog, cat, rat^23^ and mouse^19,24^. Price et al.^26^, however, found that NKCC1 mRNA expression is largely confined to small and medium diameter DRG neurons showing positive immunostaining for peripherin, CGRP or TRPV1. In contrast to the well-documented expression of NKCC1 in DRG neurons, NKCC1 protein has not yet been localized to terminals of primary sensory afferents in the spinal cord. Here, we demonstrated robust immunostaining in the axon terminals of CGRP-containing peptidergic nociceptive primary afferents in laminae I–IIo of the superficial spinal dorsal horn. The heavy staining of CGRP-IR axon terminals validates our other novel finding, namely that virtually all axon terminals of the IB4-binding nonpeptidergic nociceptive primary afferents were negative for NKCC1. Seemingly, this is in conflict with the results of those who found NKCC1 expression in all DRG neurons^19,23,24^. One may assume, however, that although NKCC1 is expressed in IB-4 binding DRG neurons, it is not transported from the cell bodies to the spinal axon terminals, similar to some ion channels that are expressed in the cell bodies of DRG neurons but missing from their central axon terminals^45,46^.

NKCC1 protein and mRNA have already been found in the somata of some neurons in the superficial spinal dorsal horn^22,26^. In the present paper, we extended these previous reports with new findings. On the one hand, our colocalization studies strongly suggest that the cell bodies and dendrites of neurons the axon terminals of which express NKCC1 in the superficial spinal dorsal horn should be located almost exclusively in lamina IIi and deeper layers of the dorsal horn, reinforcing the notion that there are neurons in the spinal dorsal horn that have their cell bodies and dendrites in laminae IIi, III and send their axons into laminae I–IIo^47^. These neurons could give rise to axon terminals in laminae I–II that were VGLUT2-IR or VGAT-IR and were also stained for NKCC1, representing 11.8% of VGLUT2-IR excitatory and 20.7% of VGAT-IR inhibitory axon terminals in laminae I–II. We have to add, however, that in addition to axon terminals of spinal origin, VGLUT2-IR had been detected also in some nociceptive primary afferent fibers. Some authors reported a noticeable decrease in VGLUT2 immunoreactivity in the spinal dorsal horn after dorsal rhizotomy^31^. In addition, Todd et al.^33^ showed that axon terminals of primary afferents retrogradely labeled with cholera toxin beta subunit in lamina I were positive for VGLUT2. Thus, the 11.8% proportion of VGLUT2-IR axon terminals in which we observed NKCC1 immunostaining can be partly axon terminals of intrinsic neurons, partly terminals of primary sensory neurons.

On the other hand, we identified two types of neurons in lamina IIi, PV-IR inhibitory and PKCγ-IR excitatory neurons^41–43^ the cell bodies and dendrites of which were clearly stained for NKCC1. It is important, however, to note that the intensity of NKCC1 expression was remarkably different in these two types of cells. PV-IR neurons were heavily stained whereas PKCγ-IR neurons showed only very weak staining for NKCC1. In addition, most of the NKCC1 immunostained puncta were recovered in the cytoplasm of PV-IR dendrites and cell bodies, which may have an important functional consequence (see next chapter).

Confirming earlier findings^44,48^ we also found NKCC1 immunoreactivity in spinal glial cells. Although NKCC1-IR puncta were recovered in large populations of processes of both GFAP-IR astrocytes (53.2%) and IBA1-IR microglial cells (62.9%), immunostaining for NKCC1 was very weak in both cell types, especially in microglial cells.

### Functional consideration

#### NKCC1 expression on CGRP-IR primary afferents

Some twenty years ago, it was demonstrated that the thermal nociceptive thresholds was altered in NKCC1 KO mice^19^. This finding was substantiated by several subsequent behavioral studies supporting the pro-nociceptive role of NKCC1 in persistent pain^17,49,50^, showing that the administration of the NKCC1 antagonist bumetanide has a significant antinociceptive effect^49^. It has also been demonstrated that in chronic pain conditions provoked by intradermal capsaicin injections, touch-evoked pain was reduced in NKCC1 deficient mice^17^, and this reduction could be mimicked by blocking NKCC1 via spinal application of bumetanide in wild-type animals^20,51^. These results generated the hypothesis that NKCC1 plays a role in spinal nociceptive information processing, and can be a factor that may participate in the mediation of touch-evoked pain.

A number of experimental evidence substantiates the idea that stimulation of low-threshold mechanoreceptors or their afferent fibers (Aβ) can evoke depolarization of nociceptive (C/Aδ) primary afferents^6^. It has been hypothesized that this phenomenon, known as primary afferent depolarization (PAD), is generated by spinal interneurons that provide interactions between Aβ and C/Aδ primary afferents. According to the most widely accepted notion, activities of Aβ fibers excite inhibitory GABAergic neurons in the spinal dorsal horn. The activated inhibitory neurons make contacts with the spinal terminals of C/Aδ fibers through axo-axonic synapses. Then, GABA released in these synapses depolarizes the terminals of C/Aδ fibers through the activation of their GABA_A_ receptors^6,52–54^. The administration of bumetanide onto the spinal cord reduces PAD^51^, suggesting that NKCC1 is involved in the generation of PAD evoked by GABA release^54^.

Thus, Aβ-fiber-mediated touch-evoked allodynia and the generation of PAD can be based on the same NKCC1-dependent synaptic mechanism^6,16,21,54^. Our present results support this earlier postulation. However, on the basis of our observations, we have to add that it is likely that PAD can be evoked in peptidergic (CGRP-IR), but not in nonpeptidergic (IB4-binding) nociceptive primary afferents. Almost all CGRP-IR axon terminals (94.45 ± 0.43%) showed strong immunostaining for NKCC1; thus, their intra-axonal Cl^−^ concentration can be so high that the activation of their GABA_A_ receptors may evoke membrane depolarization and consecutive PAD. In contrast, due to the very low level, virtual lack of NKCC1 expression, the intra-axonal Cl^−^ concentration in nonpeptidergic (IB4-binding) nociceptive primary afferents can be so low that the activation of their GABA_A_ receptors may mediate inward (hyperpolarizing) Cl^−^ currents, which can also lead to the inhibition of the postsynaptic neuronal activity, but cannot generate PAD.

It must be emphasized, however, that this study investigated naive animals, in which PAD results in presynaptic inhibition of nociceptive primary afferents^52,55^ and therefore attenuates acute pain. Under chronic pain conditions, however, both NKCC1 expression and intracellular chloride concentration may increase in the nociceptive axon terminals, as has already been observed in DRG neurons^21,24,25,56^. A Western blot study by Galan and Cervero^21^ reinforces this notion. They found that the quantity of NKCC1 protein increased by 50% in the plasma membrane prepared from the lumbosacral spinal cord following intracolonic installation of capsaicin in mice. As a consequence of the increased NKCC1 expression, the excitability of C/Aδ primary afferent terminals may increase to such an extent that the GABA_A_ receptor-mediated PAD can be so excessive that it may generate action potentials. The evoked action potentials of primary afferents can activate spinal neurons, resulting in touch-evoked pain (allodynia)^6,16,18,57^. The findings that NKCC1 KO mice display^17^ and spinal application of bumetanide results in^18^ reduced touch-evoked allodynia reinforces this hypothesis. According to our results, it is tempting to assume that touch-evoked allodynia can be more characteristic of those chronic pain states that are mediated by CGRP-IR peptidergic C/Aδ nociceptive primary afferents. Reinforcing this notion, touch-evoked allodynia has been reported in most cases in chronic pain conditions evoked by thermal, chemical or neuroinflammatory stimuli^6,16–21,51,58^, which are conducted by peptidergic primary afferents.

#### NKCC1 expression in PV-IR inhibitory neurons in lamina IIi

Studies of pharmacological blockage of inhibition in the spinal dorsal horn have revealed a novel polysynaptic excitatory drive from laminae IIi–III to lamina I^59–64^. It conducts low-threshold Aβ fiber input from laminae IIi–III onto lamina I nociceptive neurons^47^, resulting in the generation of behavioral allodynia^62^. Under control conditions, this ventral-to-dorsal excitatory pathway is blocked by tonic inhibition^65^. However, in chronic pain, the inhibition is downregulated, which enables Aβ-fiber-mediated innocuous mechanical inputs to activate nociceptive projection neurons^42,59,66^. PV-IR inhibitory neurons in lamina IIi seem to be important constituents of neural circuits regulating the transmission of Aβ fiber activities to nociceptive specific neurons in lamina I^42,44^. They receive monosynaptic inputs from Aβ fibers and inhibit those excitatory neurons, which are also activated by Aβ fiber inputs and participate in the formation of the ventral-to-dorsal excitatory pathway^41,43^. In chronic pain conditions, the excitability of PV-IR neurons is reduced^41^, and the loss of inhibition can open up the gate for the Aβ fiber polysynaptic activation of the pain pathway.

It is one of our most interesting findings that the cytoplasm of PV-IR neurons is loaded with NKCC1 but its expression in the cell membrane is limited. It is likely that cytoplasmic NKCC1 cannot influence the intracellular chloride concentration. But then, why does NKCC1 accumulate in the cytoplasm of PV-IR neurons? We do not know the answer to this question, but would like to put forward the notion that the cytoplasmic NKCC1 can be inserted into the cell membrane in chronic pain. If this happens, the influx of chloride into the PV-IR cells may increase. Consecutively, the excitability of PV-IR cells may decrease^67^, which may open up the gate for the flow of Aβ fiber activities through the ventral-to-dorsal excitatory pathway.

## Methods

### Animals and preparation of tissue sections

Animals were handled and tissue sections were prepared according to Antal et al.^68^ with some modifications. Experiments were carried out on 8 adult rats (Wistar-Kyoto, 250–300 g, Gödöllő, Hungary), two wild-type and two NKCC1 knockout mice^69^. All animal study protocols were approved by the Animal Care and Protection Committee at the University of Debrecen and were in accordance with the European Community Council Directives. Animals were deeply anesthetized with sodium pentobarbital (50 mg/kg, i.p.) and transcardially perfused with Tyrode’s solution (oxygenated with a mixture of 95% O_2_ and 5% CO_2_), followed by a fixative containing 4% paraformaldehyde dissolved in 0.1 M phosphate buffer (PB, pH 7.4). After transcardial fixation, the lumbar segments of the spinal cord were removed, postfixed in the original fixative for 3–4 h, and immersed in 10% and 20% sucrose dissolved in 0.1 M PB until they sank. To enhance reagent penetration the removed spinal cord was freeze-thawed in liquid nitrogen, sectioned at 50 µm on a vibratome, and extensively washed in 0.1 M PB.

### Immunohistochemistry

Single and double immunostaining were carried out by following the protocols of Hegyi et al.^70^.

#### Single immunostaining

A single immunostaining protocol was performed to study the laminar distribution of NKCC1 in rats and wild-type as well as NKCC1 knock-out^69^ mice. Free-floating sections were first incubated in mouse anti-NKCC1 antibody which was generated against a fusion protein fragment encompassing the C-terminus (S760-S1212) amino acids of the human colonic NKCC1 (diluted 1:3,000, T4 antibody, Developmental Studies Hybridoma Bank, University of Iowa, Iowa City, USA) for 48 h at 4 °C, and then transferred into biotinylated goat anti-mouse IgG (diluted 1:200; Vector Labs., Burlingame, California, USA) for 12 h at 4 °C. Thereafter, the sections were treated with an avidin biotinylated horseradish peroxidase complex (diluted 1:100, Vector Labs., Burlingame, California, USA) for 5 h at room temperature, and the immunoreaction was completed with a 3,3′-diaminobenzidine (Sigma, St. Louis, Missouri, USA) chromogen reaction. Before the antibody treatments, the sections were kept in 20% normal goat serum (Vector Labs., Burlingame, California, USA) for 50 min. Antibodies were diluted in 10 mM Tris–phosphate-buffered isotonic saline (TPBS, pH 7.4) to which 1% normal goat serum (Vector Labs., Burlingame, California, USA) was added. Sections were mounted on glass slides, dehydrated and covered with Permount neutral medium.

#### Double immunostaining

Double-immunostaining protocols were performed to study the colocalization of NKCC1 immunoreactivity with various markers of nociceptive primary afferents, axon terminals of glutamatergic and GABAergic spinal neurons, somatodendritic compartment of neurons, astrocytes and microglial cells. Free-floating sections were first incubated with a mixture of antibodies that contained mouse anti-NKCC1 (1:3,000) and one of the following antibodies: (1) guinea pig anti-CGRP (diluted 1:5,000, Peninsula Labs., San Carlos, California, USA), (2) biotinylated isolectin B4 (IB4) (1:2000, Sigma, St. Louise, Missouri, USA), (3) guinea pig anti-vesicular glutamate transporter 2 (VGLUT2) (diluted 1:2000, Millipore, Temecula, California, USA), (4) guinea pig-anti-vesicular GABA transporter (VGAT) (1:500, Synaptic System, Göttingen, Germany), (5) rabbit anti-parvalbumin (PV) (diluted 1:60,000, SWANT, Marly, Switzerland), (6) rabbit anti-PKCγ (diluted 1:2000, Abcam, Cambridge, United Kingdom), (7) guinea pig anti-GFAP (diluted 1:2000, Synaptic System, Göttingen, Germany), and (8) guinea pig anti-ionized calcium-binding adaptor 1 (IBA1) (diluted 1:500, Synaptic System, Göttingen, Germany). The sections were incubated in the primary antibody solutions for 2 days at 4 °C and were transferred for an overnight treatment into goat anti-mouse IgG conjugated with Alexa Fluor 555 (diluted 1:1,000, Molecular Probes, Eugene, Oregon, USA), which was mixed with either goat anti-guinea pig or goat anti-rabbit IgG conjugated with Alexa Fluor 488 (diluted 1:1,000, Molecular Probes, Eugene, Oregon, USA) or streptavidin conjugated with Alexa Fluor 488 (diluted 1:1,000, Molecular Probes, Eugene, Oregon, USA). Before the antibody treatments, the sections were kept in 20% normal goat serum (Vector Labs., Burlingame, California, USA) for 50 min. Antibodies were diluted in 10 mM TPBS (pH 7.4) to which 1% normal goat serum (Vector Labs., Burlingame, California, USA) was added. Sections were mounted on glass slides and covered with Vectashield (Vector Labs., Burlingame, California, USA).

### Confocal microscopy and analysis

Confocal microscopy and qualitative as well as quantitative evaluation of the data were performed according to the method of Dócs et al.^71^. Single and serial 1 µm thick optical sections were scanned with an Olympus FV1000 confocal microscope. Serial optical sections were scanned with an overlap of 0.5 µm. Scanning was carried out using a 60× oil-immersion lens (NA: 1.4). The confocal settings (laser power, confocal aperture and gain) were identical for all sections, and care was taken to ensure that there were no saturated pixels corresponding to puncta immunostained for NKCC1 and all the markers applied for the visualization of nociceptive primary afferents, axon terminals of glutamatergic and GABAergic spinal neurons, somatodendritic compartment of neurons, astrocytes and microglial cells. The scanned images were processed in Adobe Photoshop CS5 software.

The colocalization of NKCC1 with the investigated markers was quantitatively analyzed in the double-stained single 1 µm thick sections. A 10 × 10 standard square grid in which the edge length of the unit square was 5 µm was placed onto the regions of the confocal images corresponding to laminae I–II of the superficial spinal dorsal horn. The proper placement of the grid was based on the following criteria: (a) The border between the dorsal column and the dorsal horn was easily identified on the basis of the intensity of immunostaining. (b) The border between laminae II and III was approximated on the basis of previous observations^72–74^. It has been repeatedly demonstrated in ultrastructural studies that there are almost no myelinated axons in lamina II, while they are abundant in lamina III. Thus, the border between laminae II and III can be defined quite precisely in ultrastructural studies, and the thickness of laminae I-II can be measured. For this reason, immunoreactivities and colocalizations were investigated in the most superficial 150 µm thick zone of the dorsal horn that had previously been identified as a layer of the gray matter corresponding to laminae I and II in the L3–L5 segments of the spinal dorsal horn.

Profiles that showed immunoreactivity for NKCC1 over the edges of the standard grid were counted. The selected profiles immunoreactive for NKCC1 were then examined to determine whether they were also immunoreactive for axonal, neuronal or glial markers. Since the NKCC1 antibody utilized in the present study was raised against the intracellular domain of the enzyme, NKCC1 immunolabeled puncta were expected to be located within the confines of the area immunostained for the markers. Thus, to define the colocalization values, we counted only those NKCC1-immunolabeled puncta that were located within the confines of the areas immunostained for the marker. The colocalization for all investigated markers was analyzed in three animals. Three sections from each animal were randomly selected and the quantitative measurement was carried out in two regions of interest (ROIs) that were randomly selected from each section. One of the ROIs was placed in a more superficial region and the other in a deeper region of the layers stained for the neuronal or glial markers, but always within the confines of laminae I–II. Thus, the calculation of quantitative figures in each case was based on the investigation of 18 ROIs. From quantitative data obtained in the 18 ROIs, box-and-whisker plots were generated by using Origin Pro 8.0 software. The mean and standard error of the means (SEM) were also calculated for each value.

In order to substantiate the glial and neuronal localization of NKCC1, short series of confocal optical sections were also investigated in the X–Z and Y–Z projections. The X–Z and Y–Z images were drawn through points of colocalization between the two markers, and the two orthogonal views were investigated for overlap.

### Controls

The specificity of the antibody raised against NKCC1 has been extensively characterized^23,75^. It has been shown that the antibody recognizes NKCC1 protein in a wide variety of cell types^75–77^, including DRG^23^ and cortical^78^ neurons.

We further tested the specificity of the NKCC1 antibody in the spinal dorsal horn according to the protocols of Hegyi et al.^79^. (1) Free-floating sections obtained from NKCC1 knock-out^69^ and wild-type mice were immunostained according to the single immunostaining protocol described above. (2) In order to obtain a more global view about the specificity of the anti-NKCC1 antibody, Western-blot analysis was performed on rats. While the animals were deeply anesthetized with sodium pentobarbital (50 mg/kg, i.p.), the spinal dorsal horn at the level of L3-L5 lumbar segments were dissected. The dorsal horn was sonicated in 20 mM Tris lysis buffer (pH 7.4) containing the following protease inhibitors (mM): EDTA (4.0), EGTA (2.5), PMSF (0.002) benzamidine (0.013), pepstatin A (0.004), soybean trypsin inhibitor (0.001), leupeptin (0.001) and aprotinin (0.001). After removing cell debris from the sonicated samples with centrifugation (1,500 rcf for 10 min at 4 °C), the supernatant was centrifuged again (12,000 rcf for 20 min at 4 °C). The pellet was resuspended in lysis buffer containing 1% Triton X-100 and 0.1% SDS, and the samples were run on 10% SDS–polyacrylamide gels according to the method of Laemmli^80^. The separated proteins were electrophoretically transferred onto PVDF membranes (Millipore, Billerica, MA, USA), and the membranes were immunostained according to the single immunostaining protocol described above. The immunostaining revealed only one immunoreactive band corresponding to the molecular mass of the glycosylated NKCC1 protein as seen in many types of cells^75,78^.

In order to test the specificity of the immunostaining protocol, free-floating sections were incubated according to the single immunostaining protocol described above with primary antibodies omitted or replaced with 1% normal goat serum. No immunostaining was observed in these sections.

## Data Availability

All relevant data are available from the authors.
